# The Treatment of Pertussis by Inhalation

**Published:** 1874-12-15

**Authors:** 


### THE TREATMENT OF PERTUSSIS BY INHALATION.

IN the *Boston Medical Journal* dated April 20th, 1871, appeared an article by John J. Caldwell, M.D., of Brooklyn, N. Y., entitled "A new and Successful Treatment of Pertissis." The treatment recommended was the following:

℞ Fl. ext. Belladonnæ, ♏ v. to x.

Potass. Bromid., ℈i.;

Ammon. Bromid., ℈ij.;

Aquæ, ℥ij. M.

Inhale one tablespoonful in the ordinary steam atomizer.

Several successful cases were reported.

In a subsequent number of the same journal Dr. J. W. Spooner reports the following additional successful cases:

CASE I.—April 1st. A boy of fourteen has had the disease for two weeks. The cough has been severe and the whoop well marked. Vomits after nearly every meal. The next record is April 5th, which is as follows: Patient has been at the office daily and used the atomizer. His cough has been less since the first inhalation, and he has whooped but once. The vomiting has ceased, and there is present but a slight cough, which is not distressing.

CASES II. and III. were two children (brother and sister) aged fifteen and twelve. Well-marked symptoms of whooping-cough had been present for two weeks. The same remedy was used for four days, under my supervision, with decided abatement of symptoms. As they were improving, I lent them a hand atomizer, which I afterwards understood they used only for a day or two. The cough lingered for several weeks in both cases, although the whoop was never well marked after the use of the atomizer. In fact, during the latter period, the disease seemed to be a simple bronchitis and nasal catarrh, the result of a series of colds, as the patients were very imprudent.

CASE IV.—A child of three years had a cough, with febrile symptoms for ten days. Yesterday, for the first time, had a decided whoop. Vomited every meal to day. Face is swollen, eyes congested, and, this morning, lids adhered from excessive secretion. The atomizer was used twice daily. Improvement commenced at once. From that date there was no vomiting, the countenance resumed a natural appearance, and at the close of the week the whoop had ceased, and in less than a fortnight not the least trace of the disease was present.

This, then, is the result of my treatment of pertussis by inhalation. When the disease is at all severe, I use the atomizer twice daily until the urgency of the symptoms is relieved, and then continue it once daily until the cough has entirely disappeared. In some cases I have somewhat varied the proportion of the ingredients, but have made no essential departure from the formula given.

